# From Traditional to Omics-Driven: Emerging Strategies for Isolation, Cultivation, and Identification of Plant Endophytes

**DOI:** 10.3390/plants15142118

**Published:** 2026-07-09

**Authors:** Xinting Chen, Jiamin Wang, Liang Tang, Zixin Zeng, Di Gao, Yuanqi Yi, Lin Qin, Yunhua Xiao, Hua Yang, Bo Yang

**Affiliations:** 1 College of Bioscience and Biotechnology, Hunan Agricultural University, Changsha 410128, China; 2 Yuelushan Laboratory, Changsha 410128, China

**Keywords:** plant endophytes, omics methods, isolation, cultivation, identification

## Abstract

Plant endophytes can regulate host plant growth, improve stress resistance, and facilitate the biosynthesis of secondary metabolites, with great research value and application potential. However, traditional approaches for the isolation, cultivation and identification of plant endophytes are constrained by low culturability, limited species diversity, and loss of their original ecological functions inside host tissues. In recent years, integrated multi-omics strategies combining metagenomics, metatranscriptomics, and metaproteomics have exhibited the greatest potential to mitigate culturability limitations by enabling genome-guided targeted strain isolation and in situ functional activity profiling, among which the cultivation and targeted isolation of endophytes benefit most from omics integration. These approaches drive a paradigm shift from conventional blind screening to precise targeted isolation, and from generic medium culture to omics-guided rational cultivation, greatly improving the accuracy of strain identification and functional characterization. Nevertheless, current omics-based strategies still face inherent limitations including high experimental costs, complex operational procedures, and challenging data interpretation. The most critical future direction lies in establishing standardized experimental protocols and shared resource databases, combined with microfluidic platforms and artificial intelligence-assisted bioinformatics analysis, to address the core bottlenecks restricting endophyte isolation, cultivation and identification. This review is the first to systematically summarize research progress on traditional approaches, omics technologies and emerging strategies for plant endophyte isolation, cultivation and identification, highlights prevailing challenges and developmental trends in this field, and provides methodological references for the efficient exploitation and sustainable utilization of plant endophyte resources.

## 1. Introduction

Plant endophytes refer to microorganisms that colonize the tissues of healthy plants without causing harm to the host, including bacteria, fungi, and actinobacteria, and represent a crucial component of plant-associated microbial resources [1]. The biological functions of plant endophytes have garnered widespread academic attention, as they play key roles in promoting plant growth, enhancing stress resistance, and regulating host metabolism [2]. However, the functions of many strains remain insufficiently explored [3], making the development and utilization of endophytic microbial resources challenging. Therefore, the isolation, cultivation, and identification of plant endophytes are widely regarded as crucial foundational steps for conducting functional analysis and studies exploring underlying functional mechanisms.

Although traditional research methods have established a mature technical system, they are constrained by limitations such as low isolation efficiency, a limited range of cultivable microbial species, and low identification accuracy [2,4]. These bottlenecks make it difficult to meet the growing demands for the in-depth exploration of endophytic microbial resources. In recent years, omics technologies have increasingly become essential tools for studying the complex biological characteristics of plant endophytes. Guided by omics approaches, new research paradigms have been developed to enhance the cultivability of endophytes and integrate accurate identification methods. These advancements provide new insights and support for the deeper exploration of plant endophytes, shifting from community structure analysis to the elucidation of functional interactions. Single-omics technologies, including genomics, transcriptomics, metabolomics, and proteomics, are generally fundamental approaches that allow for the analysis of endophyte characteristics from multiple levels, such as genetics, gene expression, protein, and metabolism. On this basis, multi-omics research strategies combining metagenomics, metatranscriptomics, and metabolomics are employed to characterize endophytic microbial community structures, offering a comprehensive analysis of the metabolic networks, genetic backgrounds, and expression regulation patterns of plant endophytes [5,6]. In particular, metabolomics can unravel metabolic dependencies among endophytes and clarify plant–endophyte chemical crosstalk [7]. Furthermore, emerging omics technologies, such as single-cell omics and spatial omics, provide new perspectives for the refined and precise study of plant endophytes [8,9]. Among spatial omics tools, MALDI-MSI has been widely applied to in situ localization of metabolites secreted by endophytes, while spatial transcriptomics maps microscale symbiotic niches. In contrast, single-cell omics applications for general endophytes remain scarce, with most studies confined to mycorrhizal and nodule symbiosis [10].

**Figure 1:**
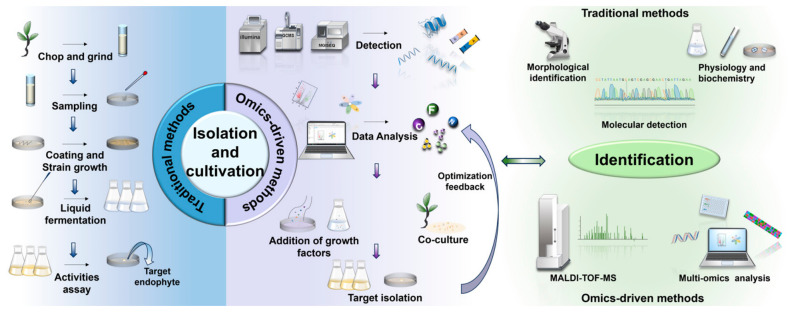
Overview of isolation, cultivation, and identification methods for plant endophytes. Blue region: stepwise workflow of traditional isolation and cultivation methods. Purple region: sequential omics-driven workflow from multi-omics detection and data analysis to optimization feedback and targeted isolation. Green region: identification methods (upper: traditional; lower: omics-driven). Arrows indicate workflow direction.

The innovative application of omics technologies has provided new perspectives for analyzing the growth, metabolism, and interaction mechanisms of plant endophytes, offering strong technical support for targeted isolation, efficient cultivation, and accurate identification of these microbes. Xiong et al. used proteomics and transcriptomics to deeply explore the interaction mechanisms between the rice endophyte *Streptomyces hygroscopicus* OsiSh-2 and its host [11]. Li et al. integrated multiple omics technologies to successfully isolate nitrogen-fixing endophytes from *Miscanthus* [12]. Deciphering these interaction mechanisms reveals the in situ nutritional demands of strains, which facilitates medium formulation and enhances endophyte culturability. The innovation of omics technologies has facilitated the development of a technical system for targeted screening, optimized cultivation design, and precise species and functional identification of plant endophytes. This has provided key technical support for the exploration and utilization of endophytic microbial resources, while also clarifying future research directions. The core technological processes of isolation, cultivation, and identification using traditional methods and omics technologies are illustrated in Figure 1.

This paper aims to provide a comprehensive review of the technical advances in the isolation, cultivation, and identification of plant endophytes. To the best of our knowledge, this work is the first to systematically synthesize and compare traditional approaches, omics technologies, and emerging strategies along the full technical pipeline for the isolation, cultivation, and identification of plant endophytes. It outlines the optimization strategies for these techniques in the field, discusses future development directions and challenges, and aims to offer technical insights for the exploration and development of plant endophyte resources.

## 2. Plant Endophytes

Plant endophytes exhibit high species diversity, primarily including bacteria (including actinobacteria) and fungi, as well as archaea and protists [2,13,14]. Among them, the primary bacterial groups include common genera such as *Bacillus*, *Streptomyces*, *Pseudomonas*, and *Rhizobium* [15]. The main fungal endophytes consist of genera such as *Colletotrichum*, *Aspergillus*, *Alternaria*, and *Nigrospora* [16]. In addition, some microalgae and viruses are also classified as endophytes, yet archaea, protists, microalgae and viruses receive far less research focus compared with bacteria and fungi [17]. Plant endophytes exert diverse biological functions, including improving host stress tolerance and regulating ecological processes. Endophytes enhance plant growth and tolerance to biotic and abiotic stresses via nutrient activation, hormone regulation and immune modulation [18,19]. They also participate in ecological nutrient cycling through hyphal networks and metabolic pathways, facilitating soil nutrient activation and supporting the restoration of degraded grassland ecosystems [20,21]. Given their broad physiological and ecological capabilities, efficient pipelines for isolating, culturing and accurately identifying endophytes must be established to tap into the potential of these valuable functional strains.

In recent years, plant endophytes have become a research hotspot in botany, microbiology, ecology and related interdisciplinary fields due to their diverse functions and broad application prospects [22,23]. Endophytes are deemed critical for developing biofertilizers and biological control strategies in agriculture [24]. The sugarcane-derived endophyte *Serratia marcescens* SM exhibits notable insecticidal activity [25]. Endophytes are increasingly recognized as an important source of natural bioactive compounds, and have the capacity to efficiently produce medicinal metabolites such as paclitaxel, vincristine and camptothecin [26]. They can also improve rhizosphere microenvironments and help enhance the cadmium phytoremediation capacity of hyperaccumulator plants [27].

Plants and endophytes have formed stable obligate symbiosis over long-term coevolution, which represents the distinctive natural history of the endophytic microbiome. Endophytes colonize plant tissues through vertical seed transmission and environmental vector-mediated horizontal colonization, assembling intricate communities composed of dominant core strains and abundant low-abundance rare strains [2,28]. Most endophytes develop stringent nutritional requirements during long-term symbiosis with host plants, such that only a small fraction can be recovered via artificial cultivation. Furthermore, closely related strains show minimal divergence in morphological features and conventional molecular markers; traditional identification approaches cannot resolve species boundaries, and evolutionary clues for undescribed novel lineages remain insufficient [16]. Although endophytes hold broad potential in agriculture, pharmacy and environmental restoration, their in-depth research and industrial use are greatly limited by traditional isolation, cultivation and identification technologies.

## 3. Traditional Methods

### 3.1. Isolation: The Dilemma of Precision and the Loss of Endophytic Microbial Diversity

The isolation of traditional plant endophytes mainly includes plant tissue selection, surface sterilization, tissue processing, strain isolation and purification [29]. Surface sterilization is widely regarded as fundamental to the successful isolation of endophytes. However, traditional surface sterilization techniques often face a core bottleneck of surface sterilization bias: the dual dilemma of over-sterilization and insufficient sterilization, both of which can compromise the effectiveness of the isolation process. Chemical surface sterilization methods typically combine 75% ethanol with suitable disinfectants [30]. However, disinfectant concentration and sterilization duration are hard to calibrate, easily leading to incomplete surface decontamination or over-sterilization. In studies on tea plants, high sodium hypochlorite concentrations reduce leaf endophyte diversity yet maintain stem bacterial richness, demonstrating varying tissue tolerances to disinfection and the necessity of tissue-specific disinfection strategies [31,32]. In addition, host-tissue specificity has been widely reported to significantly shape endophytic community composition, with strong effects of host plant species, genotype, tissue type and developmental stage. Differences in host genetics and tissue microhabitats select different microbial populations, and community composition changes continuously during plant growth. These variations result in highly variable recovery rates of culturable strains, even under the same isolation conditions [33]. The resulting large variations in endophyte richness and abundance may further determine suitable surface sterilization intensity, isolation strategies and culture conditions.

After surface sterilization of the explants, the selection of an appropriate endophyte isolation method generally depends on the characteristics of the host plant, the type of target strains, and the research objectives. Traditional endophyte isolation methods are mainly based on two strategies: the direct plating of small surface-sterilized plant explant segments, with pure single colonies isolated and purified as microorganisms grow at the tissue edges; the coating of agar plates with homogenates of ground surface-sterilized plant tissues, with pure single colonies obtained via the dilution spread plate method [34,35]. These methods have remained classic in the field of plant endophyte research due to their simplicity and wide applicability. The blue area in Figure 1 illustrates the operational steps involved in the traditional tissue homogenization method for isolating plant endophytes. However, these methods also have several limitations, such as poor targeting and susceptibility to contamination, which may significantly hinder the comprehensive development and utilization of plant endophyte resources [16,36]. Traditional isolation methods lack targeted isolation strategies and rely solely on conventional protocols, which may limit the efficient recovery of low-abundance and difficult-to-culture plant endophytes. Metabarcoding detected 5.8-fold more endophytic taxa (*n* = 88) than PDA plate isolation (*n* = 15); more than 90% of these taxa were rare low-abundance fungal lineages that failed to grow on standard artificial media [37]. This is regarded as one of the key reasons why such methods fail to fully represent the authentic composition of plant endophyte communities.

### 3.2. Cultivation: Limitations of Culture Medium and Lack of Microenvironment Simulation

Traditional cultivation of plant endophytes commonly employs standard media such as LB, PDA, MS, and NB. However, these media present inherent culture-medium bias and two critical limitations that lead to poor culturability: nutritional dependence and signaling dependence [4]. High-nutrient media strengthen resource competition and suppress oligotrophic endophytes [38]. Standard media are rich in carbon and nitrogen sources and tend to favor the growth of fast-growing endophytes. However, they are less effective at cultivating specific endophytes that thrive in low-nutrient environments. Some endophytes isolated from tomato seeds, including *Kosakonia*, *Ralstonia*, and *Sphingobium*, show limited growth on standard media but can be effectively restored by supplementing tomato tissue extract [39]. This indicates that standard media may lack essential nutrients. For such nutritionally dependent endophytes, culturability can be improved by optimizing medium components or adding specific nutrients.

Additionally, many endophytes rely on symbiotic relationships with their host plants to obtain nutrients or growth signaling molecules, which makes their cultivation more challenging under standard conditions. Dormant propagules of endophytes require plant signals to initiate germination, and nutrient supplementation alone cannot break dormancy [39]. Studies have shown that ethylene and abscisic acid secreted by sugarcane can promote and regulate the colonization of endophytic nitrogen-fixing bacteria [40]. Studies have also confirmed that adding plant tissue extracts to the culture medium can effectively improve the isolation and regrowth efficiency of recalcitrant endophytes [41]. This supports the idea that the growth of some endophytes requires signaling molecules for regulation. Therefore, incorporating plant-derived signaling compounds into standard culture media is likely to satisfy the growth regulatory needs of these endophytes.

Furthermore, loss of symbiotic function during in vitro cultivation is another critical bottleneck. Endophytes may gradually lose their essential interactive functions with plants when cultured in the laboratory [42]. The *Macrophomina phaseolina* F80 endophytic fungus from *Arabidopsis* roots is unable to convert and produce iron-chelated feruloyl-coumarin in the absence of coumarin secreted by the host plant, thereby losing its core function in mediating plant iron nutrition [43]. Traditional culture conditions often fail to replicate the microenvironment and symbiotic characteristics inside plant tissues. Owing to their simplistic nutrient composition and lack of host signaling molecules, these conditions can greatly restrict the resuscitation and normal growth of recalcitrant endophytes. Although traditional cultivation techniques are well established and easy to operate, they recover as little as 1% of total endophyte diversity, meaning over 99% of endophytes cannot be captured via standard culture approaches [44]. These methods are limited by low cultivability and the loss of in situ functionality, which may significantly hinder the in-depth exploration of plant endophyte resources.

### 3.3. Identification Technology: Low Resolution and Incomplete Database

Traditional endophyte identification methods are primarily based on morphological identification, physiological and biochemical tests, and early molecular techniques (Figure 1) [4]. This system is intuitive and easy to operate, allowing preliminary classification of strains based on colony, spore morphology, and structural features. However, phenotypic identification presents critical limitations including high subjectivity and frequent taxonomic misclassification, leading to low taxonomic resolution in traditional phenotypic identification methods [45,46,47].

The emergence of early molecular identification techniques improved accuracy to some extent; however, these methods, which rely on sequence conservancy and polymorphism, still suffer from insufficient resolution. The commonly used 16S rRNA for endophyte identification can suffer from high sequence similarity among closely related species and insufficient resolution at the species level [48]. Studies have shown that the classification results based on the 16S rRNA gene exhibit significant discrepancies when compared to those derived from genomic evolutionary relationships [49]. As the universal DNA barcode for fungi, ITS exhibits insufficient species-level resolution for certain plant-associated and endophytic fungal taxa. Furthermore, marked length variation and pronounced primer amplification bias may further compromise the reliability of fungal molecular identification [50,51]. Beyond the above two ribosomal markers, various alternative molecular markers are available for endophyte identification, including single-gene loci, functional genes and fingerprinting profiles [36]. These markers perform well for preliminary classification at the genus level. However, long-term plant–endophyte coevolution creates highly conserved gene sequences among closely related strains, so such markers lack sufficient polymorphic sites to distinguish cryptic species within the same genus.

With respect to database incompleteness, most of the endophyte gene sequences currently available in public databases belong to common bacterial phyla, with sequences from rare or habitat-specific endophytes being incomplete or even absent. Some sequences lack reference strains to verify their taxonomic accuracy, and insufficient sequence coverage may significantly impact the accuracy of annotations [2,52]. Since public databases lack type-strain reference sequences for endophytic *Mollisia*, rare endophytic OTUs of this group can only be roughly assigned, failing to discriminate closely related species [53]. Overall, identification relying on phenotypic traits and single conserved genes has inherent limitations, and it is difficult to achieve accurate taxonomic delineation of closely related and rare endophyte species.

In summary, traditional research paradigms face issues such as the lack of standardized processes, low cultivability of endophytes, and difficulty in analyzing in situ characteristics, which have widely become key bottlenecks limiting the research and development of endophytes. Therefore, there is a growing need to build upon traditional research techniques and integrate powerful, culture-independent multi-omics-driven technologies, offering a crucial direction for overcoming the limitations in endophyte research. To systematically analyze the technical characteristics of traditional methods, omics-driven technologies, and cutting-edge techniques in the isolation, cultivation, and identification of plant endophytes, the related technological systems are summarized in Table 1.

## 4. Omics-Driven Methods

**Figure 2:**
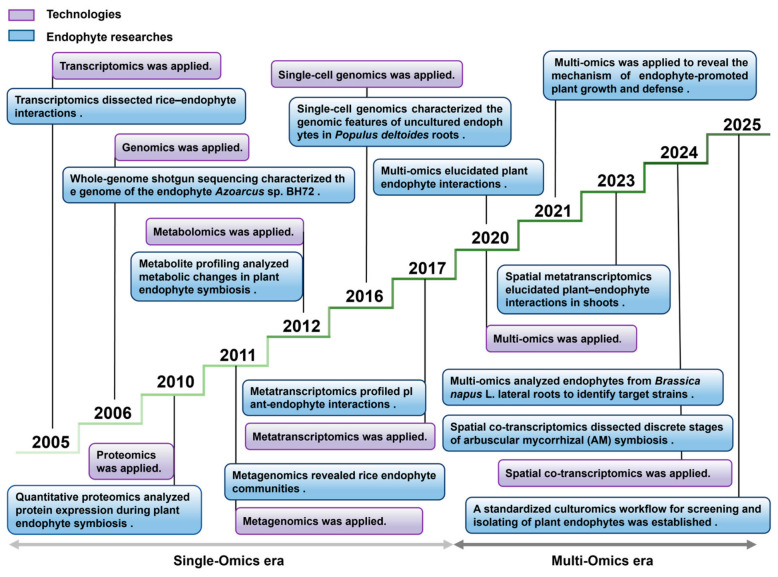
Timeline of omics applications in plant endophyte research, separated into the Single-Omics era (2005–2017) and Multi-Omics era (2017–2025) by horizontal arrows. Purple boxes mark the introduction of key omics technologies, and blue boxes show their corresponding representative applications in endophyte studies. All technological milestones and research cases presented in this figure are compiled from references [11,60,63,66,67,68,69,70,71,72,73,74,75].

Omics technologies have provided new strategies for the isolation, cultivation, and identification of plant endophytes. Currently, numerous studies have indicated that research on plant endophytes has gradually shifted from traditional methods to omics-driven approaches. Technologies such as metagenomics, metatranscriptomics, and metabolomics have been widely applied in endophyte studies, with their development timeline illustrated in Figure 2. Various omics technologies have demonstrated great potential in plants such as rice, wheat, and sugarcane. For example, Walitang et al. used proteomics to reveal the interaction mechanisms between rice and its endophytic microbes, as well as their stress response mechanisms [58]. Xia et al. employed metagenomics and metabolomics to uncover the interaction and defense mechanisms between maize and its endophytic microbes [65]. Elucidating plant–endophyte interactions and stress defense mechanisms can provide theoretical references and research insights for the targeted isolation, cultivation optimization, and functional characterization of related endophytes. A summary of the applications of relevant omics technologies in plant endophytes is provided in Table 2. Multi-omics technologies have increasingly emerged as the mainstream in plant endophyte research, addressing key limitations of traditional approaches.

### 4.1. Targeted Isolation: From Blind Screening to Precise Screening

Traditional methods for isolating plant endophytes are typically based on random screening and cultivation dependent approaches, which are not only inefficient but also prone to losing key endophytes involved in host interactions. Omics-driven targeted strain selection involves extracting and sequencing the total DNA of the host plant’s endophytic microbial community, allowing for the identification of relevant core genes. The workflow proceeds sequentially: raw reads are assembled and binned into MAGs, followed by dRep dereplication, quality evaluation, and functional annotation via KEGG and COG databases; SIP and FISH further assist in screening metabolically active endophytes in planta [85,86]. This is followed by constructing a candidate strain library and selecting core candidate strains based on omics data. The quality of recovered MAGs is evaluated following MIMAG benchmarks, which define medium-quality genomes with completeness ≥50% and contamination <10% [87]. Considering the frequent fragmentation of plant endophyte genomes, a higher completeness cutoff should be applied. Next, qualified MAGs are utilized to pinpoint functional genes, which guide subsequent targeted isolation and experimental validation to recover viable target strains, improving the efficiency of isolating desired strains.

The metagenomic-based functional prediction isolation strategy involves high-throughput sequencing of plant endophyte communities, combined with binning techniques to obtain high-quality MAGs of endophytes. Key features of this method are listed in Table 1. This approach constructs a non-redundant gene set of the endophytic microbiome and identifies key functional sequences. Carrión et al. sequenced the metagenome of beetroot root endophytes and applied binning techniques to obtain high-quality MAGs [88]. They successfully identified functional genes related to disease resistance and biosynthesis, and isolated endophytic strains with disease-resistant capabilities. Nevertheless, whether the functional genes of isolated endophytes are expressed requires further validation through metatranscriptomics. Metatranscriptomics, using the DESeq2 differential analysis tool combined with the apeglm algorithm to correct log2FC values, identified significant upregulation of target genes involved in root microbiome phosphorus transport and energy metabolism. These gene expressions were further validated through targeted experiments, providing technical support for the validation of active gene expression in microbial communities [89].

For some hard-to-isolate microorganisms, single-cell genomics focuses on culturing individual endophytic cells, overcoming the limitations of cultivation-dependent methods. SM et al. successfully constructed the genome of rare endophytes from the roots of poplar using single-cell genomic sequencing [71], providing a methodological basis for isolating rare endophytes. Cross et al. [90] proposed a reverse genomics isolation technique, where relevant targets are selected based on single-cell genomic or metagenomic data. Specific antibodies are then prepared, fluorescently labeled, and combined with flow cytometry for targeted isolation of the desired microorganisms. This strategy provides a practical paradigm for translating sequence information directly into culturable target strains. Even so, single-cell sequencing is hampered by uneven whole-genome amplification, which introduces obvious sequence bias and often results in incomplete genome assembly [71]. Furthermore, tools like dRep, which perform dereplication, can classify genomes and eliminate redundant sequences, thereby removing interference from duplicate sequences. This may significantly improve the reliability of functional gene and metabolic pathway annotations [91], providing more robust molecular support for the precise isolation of endophytes.

After identifying the core functional gene clusters, further annotation of the target genes is performed using databases such as KEGG and COG, allowing for the clarification of the metabolic potential of the target endophytes. Wang et al. [92] performed KEGG pathway analysis on the whole genome of the potato endophyte Q2H1 and identified genes related to siderophores and auxin synthesis. By integrating KEGG pathways, they further annotated and categorized its functional roles, providing precise targets for subsequent functional validation. Metagenomic high-throughput sequencing combined with binning techniques can identify key gene clusters, while metatranscriptomics validates the expression of functional genes. dRep ensures the reliability of annotations, and KEGG/COG analyses clarify metabolic potential, all of which can support the precise isolation of endophytes. Overall, the combination of omics technologies and bioinformatics tools holds the potential to establish a complete technical workflow for precise isolation of endophytes.

However, in situ functional verification of endophytes still requires further investigation. Stable isotope probing (SIP) and fluorescence in situ hybridization (FISH) are generally regarded as effective in situ functional verification techniques for validating the functions of endophytes [93,94]. By integrating SIP-based functional activity identification with FISH-derived spatial distribution data, specific primers can be designed to establish a high-throughput PCR pre-screening system. This approach allows for the rapid identification of positive clones from plant tissues, thereby enhancing the throughput of functional endophyte isolation. In studies focusing on *Miscanthus sinensis*, the combination of ^15^N_2_-labeled DNA-SIP technology with FISH successfully localized the ecological niche of active nitrogen-fixing bacteria [12], thereby accelerating the screening efficiency of target endophytes. The semi-automated high-throughput stable isotope technology developed by Nuccio et al. significantly enhances sample throughput and experimental reproducibility [95]. This targeted localization screening approach, originally applied to arbuscular mycorrhizal fungi, can also be adapted and applied to the isolation of plant endophytes. Thus, the integration of in situ validation techniques and omics data helps further refine the system for screening and localization of functional endophytes.

### 4.2. Cultivation Optimization: From General Cultivation to Rational Design Cultivation

The core prerequisite for the successful cultivation of endophytes largely lies in providing appropriate nutrition and a suitable growth microenvironment. Endophytic community structure is generally shaped by host plant species, genotype, tissue type and developmental stage, which leads to differences in nutritional requirements and growth characteristics of strains. Such host-related background characteristics should also be considered when designing culture conditions based on omics data. Unlike traditional general-purpose cultivation methods, the application of omics technologies can reveal the nutritional requirements [96], interaction mechanisms with the host plant [97], and environmental adaptation mechanisms [98] of endophytes, facilitating the shift from cultivation-dependent to design-driven cultivation. The growth factors provided by the host plant’s internal environment to endophytes can be predicted through omics data analysis, including metagenomics, metatranscriptomics, proteomics, and metabolomics. This allows for the design of growth nutrients and environments that closely resemble their natural endophytic conditions, which can enhance the cultivability and diversity of plant endophytes.

Omics technologies provide a scientific methodology for designing the cultivation of endophytes. Before medium formulation, omics annotations predict metabolic pathways, and substrate control cultures coupled with metabolite quantification validate these pathways to guide medium optimization [99]. By using metagenomic sequencing, all microbial DNA from plant tissues can be directly extracted, allowing for the assembly of metagenomes and the genomes of hard-to-culture endophytes. By using binning techniques to analyze sequence information, the key nutritional carbon sources, nitrogen sources, and growth factors required for these microorganisms can be inferred [100]. This approach enables the design of specific media, potentially overcoming the cultivation bottlenecks of hard-to-culture endophytes. Prasannakumar et al. [101] used shotgun sequencing to analyze the functional genes of the sorghum endophytic community. By integrating KEGG pathway annotations, they identified the specific carbon and nitrogen requirements of the microorganisms, providing a basis for optimizing culture media for specific endophytes. Li et al. used binning techniques to assemble a genomic draft from *Miscanthus* roots, identifying *nifH* (nitrogenase gene), *czcA* (heavy metal resistance gene), and *acdS* (plant growth-promoting gene) [12]. They further validated the metabolic activity of the endophytes through DNA-SIP, successfully isolating nitrogen-fixing endophytes.

The accuracy of genome binning has improved the targeted identification of microorganisms from host plants and enhanced the efficiency of microbial isolation and cultivation. Sieber et al. [102] proposed the DAS tool, which incorporates dereplication, aggregation, and scoring strategies. This tool, when applied to microbial genome analysis, yields a significantly higher number of endophyte types compared to other tools. It has reported the highest number of nearly complete and draft-level bins in human gut, oil seep, and soil samples. By integrating the advantages of multiple algorithms, the DAS tool achieves precise optimization of metagenomic binning, providing critical technical support for high-quality identification of metagenomic drafts. Omics technologies enable the prediction of strain nutritional preferences at the genomic level, thereby greatly reducing trial-and-error in medium formulation.

For symbiotic endophytes that rely on host plant secondary metabolites, metabolomics directly analyzes the metabolic products in plant tissues to identify the substances that influence the growth and metabolic activities of plant endophytes. Harbort et al. [103] found that coumarin compounds produced in *Arabidopsis* roots under iron deficiency significantly promote the growth of beneficial endophytes. Metabolomics can detect plant-microbe interactions and elucidate the key nutrients required for microbial growth. In a study on the medicinal plant *Elephantorrhiza elephantina*, RNA-seq-guided medium supplementation with 1% olive oil and 0.5% yeast extract increased the number of cultivable endophytic species from 4 to 17. Xu et al. performed transcriptomic analysis and found that hypoxia-response genes were highly induced in the symbiotic tuberoids of *Dendrobium officinale*, suggesting that endophytic fungi in the roots may exist in a low-oxygen environment [104]. This provides a basis for controlling oxygen concentration in the in vitro cultivation of endophytes. Dissection of host metabolism and environmental signals offers a critical foundation for recapitulating the native growth conditions of endophytes.

Proteomics and metaproteomics can reflect the in situ physiological status and metabolic activity of endophytes at the protein level. By identifying highly expressed functional enzymes, nutrient transporters, and signaling proteins in native environments, these approaches clarify the nutritional requirements and growth-limiting factors of endophytes, thereby providing critical support for culturomics-based high-throughput culture condition optimization and improving the culturability of recalcitrant endophytes. Yue et al. performed quantitative proteomic analysis on the wheat endophyte *Bacillus* sp. WR13, identifying upregulated proteins including siderophore synthetases, iron transporters, and iron-dependent functional enzymes, and confirming iron as a critical growth-limiting factor [105]. Li et al. used proteomics to characterize *Mycoavidus cysteinexigens*, identifying key transporters and TCA enzymes that revealed its carbon and nitrogen preferences, with nitrogen as the primary growth-limiting factor [106]. These findings indicate that proteomics facilitates the identification of nutritional limitations and targeted optimization of culture conditions, thereby enhancing the culturability of endophytes.

In addition, spatial omics enables in situ analysis of the spatial distribution and interactions between cells and molecules in tissues, preserving authentic micro-niche information. This can provide supplementary guidance for culturomics-based culture environment simulation. However, its application in plant endophytes remains limited. Serrano et al. used spatial co-transcriptomics and single-nucleus RNA sequencing to characterize the in situ spatial relationship between arbuscular mycorrhizal fungi and host roots [8]. This strategy can be applied to plant endophyte cultivation to clarify spatial interactions and support the optimization of culture systems.

Culturomics has emerged as a key strategy for design-driven cultivation of endophytes. By simulating the in situ growth environment via high-throughput methods, it can effectively improve microbial culturability and diversity [107]. However, culturomics differs from omics-guided cultivation. Culturomics aims for high-throughput strain isolation, while omics data predict microbial metabolism and nutritional needs to direct medium development. Flores-Duarte et al. [108] employed a culturomics strategy in the halophyte *Mesembryanthemum crystallinum*. Using host-derived medium to simulate the in situ growth environment and adopting a high-throughput culturomics workflow for microbial isolation, they significantly enhanced endophytic culturability and diversity, and characterized the plant growth-promoting, metal-resistant, and metabolic traits of the isolated strains. These studies indicate that genomic and transcriptomic data provide critical guidance for defining the nutritional requirements and rational media design of endophytes. Importantly, culturomics relies on multi-omics integration rather than blind trial and error, supporting the efficient development of tailored media for beneficial endophytes.

However, many uncultivable endophytes are closely linked to microbial interactions [75]. Microbial dark matter refers to the unknown microbial taxa and functional genes that are widely present in the environment, detectable by sequencing, but difficult to isolate and pure-culture using traditional laboratory methods [109]. Co-cultivation strategies are widely regarded as a crucial pathway for unlocking the microbial dark matter. By constructing artificial microbial communities to simulate natural ecological environments, co-cultivation activates silent biosynthetic gene clusters (BGCs) to produce novel metabolites, enhancing the yield of specific target metabolites and unlocking the metabolic potential of endophytes [110]. For example, in a co-culture system of hazelnut cells and the endophytic fungus *Epicoccum nigrum*, the paclitaxel yield reached 404.5 µg/L, 136.6 times higher than in monoculture [111]. Beyond traditional monoculture, co-culture has become an important supplementary technique for the targeted cultivation of endophytes. Based on intermicrobial interactions, the co-culture strategy can effectively activate the metabolic potential of endophytes, offering a novel approach to help break through the bottleneck of their unculturability.

### 4.3. Identification: Endophyte Identification and Functional Analysis

Omics technologies provide systematic and precise technical support for the identification and functional characterization of endophyte strains. They can help overcome the limitations of traditional identification methods, which are inefficient and restricted, enabling comprehensive analysis from the genetic to the metabolic level. Metagenomics, the FastANI algorithm, multilocus sequence analysis, and single-cell genomic sequencing can enable precise strain identification of endophytes. Meanwhile, metagenomics, metatranscriptomics, proteomics, and metabolomics can progressively advance functional characterization and mechanism elucidation (Figure 1). Metagenomics enables the acquisition of complete microbial genomic information. By comparing genomes, conducting phylogenetic analysis, and integrating functional specificity, it allows for precise strain-level identification of endophytes [76,112]. The high-throughput tool FastANI enables rapid and accurate analysis of endophyte genomic data. By combining the ANI ≥ 95% species delineation threshold with phylogenetic analysis, FastANI can efficiently distinguish closely related endophyte groups. While ANI ≥ 95% serves as the standard species cutoff for prokaryotes, it is limited by an 83–95% ambiguous range covering 0.21% of genomic comparisons and taxonomic exceptions like *Escherichia*-*Shigella* complexes [113,114]. Thus, it provides crucial support for precise strain identification and addresses the low computational efficiency of traditional BLAST methods (NCBI BLAST+ v2.10.0). To overcome the limitations of using a single gene for strain identification, multilocus sequence analysis (MLSA) selects 5–10 specific genes for PCR amplification and sequencing. These genes are mostly evolutionarily conserved housekeeping genes with rare horizontal gene transfer and moderate genetic polymorphism, which can reliably distinguish closely related strains. By integrating the variation information from multiple functional genes, MLSA significantly enhances the resolution of strain identification. Pusz-Bochenska et al. conducted MLSA of core genes such as *cpn60* and *tuf* in hard-to-culture plant pathogenic phytoplasmas, successfully distinguishing different pathogenic strains [115]. The rise in single-cell genomic sequencing and gene-editing-assisted validation has further enhanced the precision of strain identification, extending it to the level of individual endophytes. Lundberg et al. successfully isolated uncultivable endophytes from *Arabidopsis* roots using single-cell genomic sequencing, performing precise species classification [116]. Compared with the high throughput of FastANI and high resolution of MLSA, single-cell genome sequencing enables precise identification of unculturable endophytes. It can compensate for the inability of the two methods to analyze individual strains, and is particularly suitable for distinguishing closely related endophytes in communities.

The application of omics technologies has addressed the long-standing dilemma that the dissection of functional mechanisms in plant endophytes was heavily reliant on cultivation, driving endophyte-related research from superficial functional prediction towards precise characterization of growth and metabolic traits. Through functional annotation against databases including KEGG and COG, metagenomics allows for systematic mining of metabolic-associated genes in uncultured endophytes, prediction of their nutritional preferences such as carbon and nitrogen utilization and substance biosynthesis, and provides theoretical support for the targeted design of culture medium formulations. Tian et al., by integrating metagenomics with KEGG pathway analysis, identified numerous genes related to plant polysaccharide degradation, carbohydrate and protein metabolism, and biological nitrogen fixation in the microbial communities of tomato root nodules [117]. It can generally be concluded that the nutritional metabolic genetic features of root endophytes are highly compatible with their in situ growth niches, which can potentially be directly employed as a critical criterion for the design of in vitro isolation and culture systems. However, the accurate expression of these functional genes requires validation through combined metatranscriptomics and proteomics analysis [118], which is key to understanding the functional mechanisms.

Metatranscriptomics reflects gene expression levels. In contrast, metaproteomics enables direct capture and identification of functional proteins expressed by endophytes in situ, including metabolic enzymes, transporters, secreted proteins, and stress-responsive proteins. It reveals metabolic activity and symbiotic phenotypes at the protein level, providing key evidence for functional mechanism analysis and helping the critical leap from “gene prediction” to “functional validation” [119,120]. Bao et al. [121] applied metaproteomics to characterize functional proteins of endophytes in rice roots. They identified key enzymes involved in nitrogen fixation and methane oxidation, confirming their in situ metabolic activities. This approach can enable precise and direct determination of endophytic functions in the native host environment.

Metabolomics directly captures the metabolic state of endophytes in their native environment and under artificial culture conditions, enabling the prediction of nutritional requirements and the optimization of culture media. Profiling metabolites in host tissues or culture systems via UPLC-MS and LC-MS/MS reveals key nutrients, growth factors, and metabolic constraints, offering direct guidance for tailored medium design. Using metabolomics and proteomics, Zhao et al. profiled the metabolic traits of the wheat endophyte *Bacillus* sp. WP-6, identifying key enrichment in amino acid, porphyrin, and chlorophyll metabolism under stress conditions [122]. Such metabolic traits mirror the strain’s nutritional preference, facilitating tailored medium formulation for better isolation and cultivation. Furthermore, in situ metabolomics enables the identification of host-derived growth factors; incorporating these compounds into culture media mimics native niches and can help improve the culturability of recalcitrant endophytes.

### 4.4. Multi-Omics Integration: Enabling Precise Isolation, Cultivation and Identification

In plant endophyte research, the in-depth application of omics technologies such as metagenomics, metatranscriptomics, metabolomics, proteomics, and single-cell genomics aims to overcome the limitations of traditional methods, including low isolation efficiency, narrow cultivation range, and insufficient identification accuracy. These advancements can provide critical support for breaking through traditional bottlenecks in endophyte research. Notably, the structure and functional characteristics of endophytic communities are generally significantly influenced by host plant species, genotype, tissue type and developmental stage. Endophytes under different host backgrounds exhibit distinct biological traits, which may further increase the difficulty of comprehensive interpretation. However, single-omics technologies still face limitations, such as only being able to predict functional potential, difficulty in elucidating the gene-function-phenotype relationship, inability to dynamically monitor endophyte growth, and low identification resolution [44]. These limitations may hinder research on rare endophytes and could fail to fully meet the technical demands. Therefore, combining multiple omics technologies, supplemented with innovative techniques, is likely to offer new strategies to overcome the restrictions in plant endophyte isolation, cultivation, and identification research [123].

The efficient development of endophyte resources largely relies on the integrated application of multi-omics and traditional cultivation. Omics technologies quickly uncover functional targets, while traditional techniques optimize cultivation conditions based on omics data to obtain pure endophytes. This aligns with the logic of culturomics and metagenomics complementarity. The combination of both approaches can hold the potential to overcome the challenges of having functional targets without endophytes or having endophytes with unclear functions. It is widely regarded as a core pathway for the utilization of endophyte resources [124]. The low cultivability of endophytes has long been recognized as a challenge in microbiological research. Transcriptomics, by analyzing the gene expression profiles of uncultivable endophytes, helps identify the substances required for their growth, thereby providing targeted guidance for endophyte isolation and significantly improving isolation efficiency [107]. Bai et al., based on transcriptomic data from *Arabidopsis* roots and leaves, designed tissue-specific culture media, which increased the number of cultivable endophyte species by more than five times, enriching the diversity of endophytes [125]. Huang et al., by combining metabolomics and multi-omics analyses, discovered triterpenoid compounds specifically synthesized by plant roots, which can help promote the colonization of certain beneficial microorganisms in the roots [57]. Clarifying the key nutritional requirements of the strain provides a basis for the targeted development of specialized culture media.

**Figure 3:**
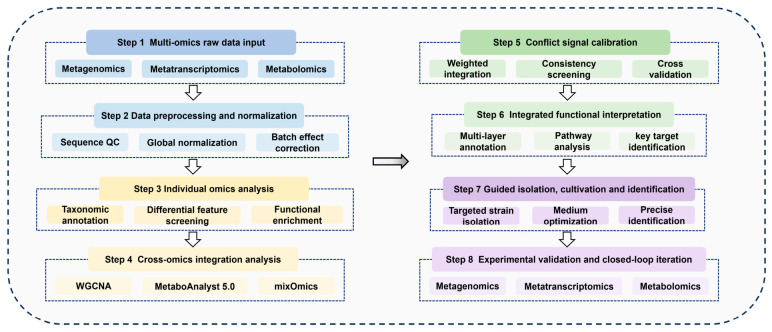
Schematic workflow illustrating the multi-omics integration strategy for guiding the isolation, cultivation, and identification of plant endophytes. The pipeline starts with multi-omics raw data (Step 1), followed by data preprocessing and normalization (Step 2), individual omics analysis (Step 3), and cross-omics integration using tools such as WGCNA, MetaboAnalyst 5.0, and mixOmics (Step 4). Conflicting signals between different omics layers are then calibrated (Step 5), and integrated functional interpretation is performed to identify key targets (Step 6). These results guide targeted strain isolation, medium optimization, and precise identification (Step 7), followed by experimental validation and closed-loop iteration (Step 8).

Integrated multi-omics analysis of plant endophytes first conducts normalization and batch-effect correction on multidimensional data from amplicon sequencing, metagenomics, metatranscriptomics, and metabolomics to ensure data comparability. Their functions, limitations, resolution, sample requirement, best use case and other relevant indicators are detailed in Table 1. Correlation analysis, multivariate statistical methods, and co-expression network analysis are then applied to reveal intrinsic associations among omics layers. Finally, functional enrichment and joint annotation are performed to achieve systematic integration and biological interpretation of the multidimensional data [126]. Lopez-Barbera et al. [127] used yeast to conduct cross-analysis integrating genomic, transcriptomic and metabolomic data for metabolic pathway verification, validating this layered integration paradigm. This strategy can also be applied to plant endophytes. However, discordant expression patterns are often observed across different omics layers including metatranscriptomics and metaproteomics, which can be corrected via statistical approaches such as weighted integration, consistency filtering and cross-validation [128]. A reasonable data correction algorithm is generally regarded as a key prerequisite to ensure that multi-omics results conform to the real physiological state of the strain. This integrated multi-omics analytical workflow is schematically illustrated in Figure 3.

Currently, bioinformatics tools and statistical frameworks including WGCNA, MetaboAnalyst 5.0, mixOmics and iOmicsPASS are generally widely applied in plant endophyte research to conduct correlative analysis of metagenomic, metatranscriptomic and metabolomic data, thereby supporting the isolation, screening, culture optimization and taxonomic identification of endophytes. Among these approaches, WGCNA, as a classic multi-omics integration algorithm, can enable the construction of gene-metabolite co-expression networks and the identification of key co-expression modules and core regulatory factors closely associated with the growth characteristics, biological functions, isolation, cultivation and taxonomic identification of plant endophytes [60]. MetaboAnalyst 5.0 supports metabolite annotation and pathway enrichment analysis, contributing to the screening of pivotal metabolic signatures linked to strain growth and functional traits [129]. The mixOmics framework adopts multivariate statistical algorithms to achieve joint dimensionality reduction, feature screening and module correlation analysis for multi-omics data, allowing researchers to mine key factors governing microbial cultivability from high-dimensional omics datasets [130]. Furthermore, iOmicsPASS realizes cross-omics integration and functional prediction at the pathway level, and can identify core regulatory units that modulate the isolation and cultivation efficiency of endophytic strains [131]. Nevertheless, the above tools have inherent limitations. WGCNA lacks reliable cross-omics integration capacity, mixOmics is unable to resolve hierarchical regulatory networks, and iOmicsPASS entails high computational costs with limited applications in endophyte research. Some tools have high requirements for data distribution characteristics and sample size. The biological interpretation of cross-omics association results still requires manual verification, and complex data analysis processes have certain demands for computing resources and bioinformatics skills.

Multi-omics technologies can cross-validate three key dimensions: genetic basis, active expression, and functional products, providing technical support for the targeted isolation of endophytes. Taking the isolation of nitrogen-fixing endophytes as an example, metagenomics is first used to predict functional genes, followed by the construction of a plant endophyte gene set using Illumina sequencing technology, and the prediction of the *nifH* gene [132]. Subsequently, metagenomic and metatranscriptomic analyses are combined to examine the expression changes in *nifH* under different low-nitrogen conditions [133]. Then, ^15^N-SIP and FISH technologies are used to localize nitrogen-fixing bacteria. For example, Wang et al. employed these techniques to identify active nitrogen-fixing bacteria in rice paddy soil and validated their distribution within root cortical cells [134]. A reasonable data correction algorithm is a key prerequisite to ensure that multi-omics results conform to the real physiological state of the strain.

Integrated multi-omics analyses enable multi-level cross-validation, overcoming the limitations of single-omics approaches. Corroboration across multi-dimensional data allows precise characterization of endophyte metabolism and growth traits, providing robust molecular support for targeted isolation, culture optimization, and species identification. The integration of multi-omics with traditional culture experiments is increasingly regarded as an important trend in plant endophyte research. Currently, databases such as GenBank, KEGG, and IMG/M have amassed a wealth of omics data on plant endophytes, providing essential support for resource exploration and functional analysis. To facilitate the subsequent implementation of omics experiments, the core databases, application areas, and website links for several omics technologies are summarized in Table 3.

## 5. Challenges and Future Directions

Although omics technologies have significantly advanced the depth of plant endophyte research, they also face numerous challenges. Lately, the cost of high-throughput sequencing has dropped dramatically. For example, metagenomics-based sample pooling strategies can further reduce the costs of sequencing and library preparation for plant endophytic communities [145]. Notably, the cost of whole-genome sequencing has decreased markedly in recent years compared with the high pricing observed a decade ago, showing a clear downward trend across mainstream sequencing platforms. However, with the exponential growth in the output of multi-omics sequencing data, subsequent bioinformatics analysis, high-performance computing resources, professional personnel, and data storage still may present high economic and technical barriers [146,147]. These may continue to pose substantial limitations for resource-limited laboratories.

The plant–endophyte system features complex compositions and diverse metabolic backgrounds, while multi-omics data are characterized by large volumes and significant dimensional heterogeneity, imposing stringent requirements for data integration and standardized analysis. Existing multi-omics analysis tools still have limitations in terms of efficiency and accuracy when handling large volumes of data. In addition, the integration and analysis of omics data lack standardized workflows. For instance, there are variations in the preprocessing methods for metabolomic data, such as those for the microbiome, between different laboratories, making effective data comparison challenging [148].

Most current plant endophyte studies rely on in vitro cultivation or tissue extraction methods, which disrupt the original spatial distribution of plant endophytes and their interactions with plant-associated microbes. This may fail to accurately reflect the functional roles of endophytes under natural conditions. To systematically analyze the spatial distribution and interactions of different plant endophytes within plant tissues, both spatial dimension and bioinformatics approaches must be employed for in-depth analysis [149]. However, this also increases the complexity of data integration and interpretation. Therefore, there is a growing need to develop more efficient, accurate, and standardized research strategies.

The establishment of standardized experimental protocols and resource databases is fundamental to advancing the field of plant endophyte research. Currently, the lack of standardized procedures greatly restricts the collaborative development of research in this area [4]. Significant differences in the methods used by different laboratories in processing tissue data have led to difficulties in making cross-comparisons of research findings [150]. Therefore, the pre-treatment of experimental materials and the establishment of standardized laboratory standard operating procedures (SOPs) are widely considered particularly crucial. The standardized SOPs should include detailed steps for plant endophyte isolation, such as sample selection, surface sterilization, sterile procedures, strain purification and separation, and strain preservation, thereby providing a solid theoretical foundation for researchers. Novak et al. [151] developed standardized SOPs for key experimental procedures, including surface sterilization, aseptic manipulation, and culture medium sterilization. Based on these protocols, five laboratories conducted 210 aseptic experimental assessments, yielding a contamination rate of <1%, which improved the reliability of the research outcomes to some extent. Zhou et al. [152] constructed the standardized sequence alignment databases reprDB and panDB. When applied to the annotation of highly complex microbial communities, the sequencing read annotation rates reached 49.7% and 75.1%, significantly improving the accuracy of microbial identification. The development of plant endophyte resource databases could draw on the construction strategies employed in these standardized databases, such as reprDB and panDB. By integrating sequence data of plant endophytes derived from the same tissues across different host plants, it becomes possible to facilitate the rapid acquisition of relevant and informative datasets [153]. However, issues related to the standardization and ownership of plant endophyte databases still require further clarification.

The innovative application of microfluidic technologies is expected to open new avenues for the cultivation and isolation of plant endophytes, thereby promoting deeper exploration in this field. Richter et al. [154] summarized the Fungi-on-a-Chip platform, which enables the precise cultivation of fungal single cells. In particular, the double emulsion droplet-based microfluidic system can establish a micro-cultivation platform that integrates internal nutrient supply, intermediate target metabolite capture, and external stable encapsulation to simulate the microenvironment within plant tissues, thereby enabling the in situ cultivation of host-dependent fungi. This technique is particularly suited for plant endophytic fungi that are difficult to recover using conventional plate culture, and can significantly improve the culturability of these recalcitrant taxa. McCully et al. [61] developed the GrowMiDE platform, which enables the efficient enrichment of rare and difficult-to-culture microorganisms and may improve the culturability of plant endophytes. This platform utilizes double emulsion microencapsulation to entrap individual microbial cells with trace nutrients, constructing a stable microscale culture environment and further enhancing the isolation efficiency of slow-growing and oligotrophic endophytes. Liu et al. [155] established a microfluidic high-throughput screening platform that enables the co-cultivation of plant endophytes and host plants. Using this system, they successfully isolated the plant endophyte *Bacillus paramycoides* (PE1), which increased the root growth rate of the hyperaccumulator plant *Phytolacca acinosa* by 54.31% and enhanced the removal efficiency of the heavy metal Cd by 55.49%. Such a setup circumvents the limitations of conventional methods that cannot simultaneously monitor plant growth and dynamic endophyte–host interactions, providing an efficient technical strategy for the targeted screening of functional endophytes.

For difficult-to-cultivate endophytes with extremely low abundance and those highly dependent on host interactions, single-cell genomics is expected to overcome the resolution limitations of conventional omics. Fluorescence-activated cell sorting (FACS) enables targeted sorting using FISH probes, as well as label-free sorting based on cellular physical properties for high-throughput identification and isolation of single cells [156]. Endophyte cultivation guided by single-cell Raman spectroscopy-sequencing combined technology is generally more efficient and targeted [157]. The integration of microfluidic single-cell cultivation with single-cell omics can encapsulate individual endophytes in picolitre-scale droplets, allowing real-time monitoring of cellular metabolic activity and precise capture of functionally active endophytes predicted by metatranscriptomics, thereby alleviating the loss of functional endophytes in traditional cultivation to a certain extent [158]. The combination of microfluidics and mass spectrometry allows rapid detection of secondary metabolites within droplets, providing important technical support for the study of microbial secondary metabolites in droplet-based systems [159]. Notably, microfluidic techniques are generally limited by complex operation and high cost, which may restrict their widespread application.

The integrative analysis of multi-omics datasets faces substantial challenges due to the large data volume and the heterogeneity in data dimensions. Against this background, artificial intelligence (AI) may provide intelligent analytical support for endophyte research [160]. Through intelligent analysis of colony phenotypic characteristics, AI-based approaches could assist the precise screening of diverse plant endophytes [161]. Diao et al. constructed an AI-RACS platform combining microfluidic trapping and machine learning to efficiently screen rare metabolically active endophytes from plant samples [162]. By integrating microfluidic technology with AI-based visual analysis, a three-pronged platform integrating static array single-cell isolation, dual deep learning-based multi-modal phenotyping and LIB contactless sorting was built for high-throughput single-cell strain screening [62]. This technological framework could be further applied to research on plant endophytes. Evangelisti et al. [63] developed AM Finder, which adopts a two-stage hierarchical recognition mechanism: CNN1 segments images into tiles to judge root infection status and quantify overall colonization levels, while CNN2 further analyses infected regions to identify intracellular fungal structures for fine qualitative analysis. It can automatically identify and quantify the colonization of arbuscular mycorrhizal fungi in plant roots. Although AM Finder was originally built exclusively for arbuscular mycorrhizal fungi, its deep-learning image recognition framework can be transferred to the morphological screening of plant endophytic colonies. In addition, AI-driven integration of multi-omics data has helped enable the establishment of a “Design–Build–Test–Learn-Predict” research framework (DBTLP), which can utilize AI for data learning and prediction, with continuous experimental feedback to retrain the model and can be further extended to predict the optimal culture conditions for endophytes and assist in medium optimization and strain screening [64]. Notably, AI analysis relies on large-scale, high-quality annotated datasets, which remain scarce in the endophyte research field. Meanwhile, the recognition accuracy of models needs to be improved in highly complex plant-microbial communities, and a gap still exists between proof-of-concept validation and reliable practical application.

The application of omics technologies is expected to promote further advances in the isolation, cultivation, and identification of plant endophytes. Building on standardized SOPs and database development, the innovative application of microfluidic technologies could facilitate omics-guided targeted isolation and screening, combined with AI-assisted techniques to integrate massive omics data analysis. Together, these strategies may drive the exploration of plant endophyte resources, help elucidate the key influencing factors of microbial cultivation and the complex mechanisms of plant-microbe interactions, and steadily advance the exploration of plant endophyte resources toward targeting, precision, and high cultivability.

## 6. Conclusions

During the isolation, cultivation, and identification of plant endophytes, several persistent bottlenecks remain, including low culturability, loss of in situ functions, and limited taxonomic resolution during identification. These challenges have long constrained the in-depth advancement of research on plant endophytes. Advances in microbial research urgently require innovative technological support. Omics-based approaches are widely regarded as providing essential technical foundations for the targeted isolation, rational cultivation design, and precise identification of plant endophytes. For instance, metagenomics enables the prediction of microbial functional genes, metabolomics facilitates the detection of microbial metabolites to clarify their nutritional requirements, and metatranscriptomics verifies the expression activity of functional genes. Meanwhile, the integration of emerging omics approaches, such as culturomics, single-cell omics, and spatial omics, may markedly improve the precision of isolation and cultivation of plant endophytes, thereby facilitating the efficient exploration of rare plant endophyte resources.

In the future, omics-driven strategies for the isolation, cultivation, and identification of plant endophytes could focus on two key directions. On the one hand, efforts might be directed toward developing cost-effective experimental workflows and integrated data analysis platforms, thereby providing an important foundation for the broader application of omics technologies. On the other hand, it is worth noting that the multidimensional integration of multi-omics technologies is likely to remain a central theme in future plant endophyte research. Coupling these approaches with techniques such as reverse genomics, microfluidic cultivation, and in situ cultivation may provide new avenues for addressing key challenges in the study of plant endophytes. For the exploration and utilization of rare and difficult-to-culture plant endophytes, future efforts may focus on the ecological characteristics of their habitats and their specific nutritional requirements, thereby facilitating the development of more mature and advanced technological frameworks.

## Figures and Tables

**Table 1:** Summary of methods for isolation, cultivation and identification of plant endophytes.

Type	Method	Function	Advantages	Limitations	Resolution	Cost	Sample Requirement	Primary Application	Required Validation	References
Traditional methods	Tissue homogenization method	Isolation	Simple operation	Low isolation efficiency, easy contamination	Low	Low	Fresh plant tissues, surface sterilization	Preliminary isolation of endophytes	Surface sterility validation	[16,52]
Traditional methods	Tissue plating method	Isolation	Simple operation	Low isolation efficiency, easy contamination	Low	Low	Fresh plant tissues, surface sterilization	Preliminary isolation of endophytes	Surface sterility validation	[16,52]
Traditional methods	Phenotypic identification	Identification	Simple operation	High subjectivity, poor reproducibility	Low	Low	Pure endophytic isolates	Genus-level preliminary identification	Not required	[16]
Traditional methods	Molecular identification	Identification	Simple operation, high versatility	Insufficient resolution	Medium	Low	Pure endophytic isolates	Accurate species-level identification	PCR quality control	[49,54]
Omics-driven methods	Metagenomics	Isolation, cultivation and identification	Target isolation	High cost, complex data analysis	High	High	Sterilized plant tissue homogenate	Comprehensive profiling of total endophyte communities	Extraction blank control and sample quality validation	[12]
Omics-driven methods	Metatranscriptomics	Isolation, cultivation and identification	Species assignment, function validation	Requires rapid RNA stabilization; susceptible to host RNA contamination during extraction	High	High	Sterilized plant tissue homogenate	Functional profiling of active endophytic communities	Extraction blank control and sample quality validation	[55]
Omics-driven methods	Metabolomics	Isolation, cultivation and identification	Accurately identify functional metabolites	Limited metabolite detection coverage	Low	High	Sterilized plant tissue homogenate; pure endophytic isolates	Metabolic profiling of endophyte-host systems	Extraction blank control and sample quality validation	[56,57]
Omics-driven methods	Metaproteomics	Isolation, cultivation and identification	Directly reflects endophytic functional activity with high resolution	Highly dependent on reference databases	High	High	Sterilized plant tissue homogenate	Functional profiling of active endophytic communities	Extraction blank control and sample quality validation	[58,59]
Omics-driven methods	Multi-omics	Isolation, cultivation and identification	Accurate mechanism dissection	Technically complex and difficult to resolve	High	High	Sterilized plant tissue homogenate; pure endophytic isolates	Integrative profiling of the endophyte-host interaction system	Extraction blank control and sample quality validation	[5,60]
Emerging methods	Double emulsion droplets	Isolation, cultivation and identification	Enrich slow growers, high throughput, viable sorting	Anaerobic dyes limited, strict anaerobes oxygen-sensitive	Medium	Medium	Sterilized fresh plant tissue homogenate; purified endophytic protein lysate	Multiplex detection of specific endophyte functional proteins	Extraction blank control and sample quality validation	[61]
Emerging methods	AI-based visual analysis	Isolation, cultivation and identification	Efficiency improvement	Limited algorithms, incomplete manual validation independence	Medium	High	Pure-cultured endophytic strains	High-throughput screening of secondary metabolite-producing endophytes	Image repeatability validation and quality control	[62]
Emerging methods	Automatic Mycorrhiza Finder	Isolation, cultivation and identification	Accurate, efficient, open-source and reproducible	Structural Limitations, Narrow Application Scope	Medium	High	Endophytic genomic sequences (MAGs)	Pre-culture virtual screening of root endophytes and mycorrhizal fungi	Result repeatability validation and quality control	[63]
Emerging methods	DBTLP	Isolation, cultivation and identification	Establishes an omics closed loop to iteratively optimize culture conditions	High labor and time costs caused by repeated rounds of experiments.	High	High	Endophytic genomic sequences (MAGs)	Iterative culture optimization for hard to cultivate plant endophytes	Repeatability validation and quality control	[64]

**Table 2:** List of omics-based investigations of endophytes from diverse plant sources.

Endophytic Taxa	Host Plants	Omics	Applications	References
*Methylobacterium oryzae* CBMB20	Rice	Proteomics	Reveal plant-microbe interaction and stress response mechanisms mediated by endophytes	[58]
*Bacillus*	Maize	Metagenomics, metabolomics	Reveal host microbe interaction and defense mechanism	[65]
*Purpureocillium lilacinum* YZ1	Wheat	Metabolomics	Characterization of antifungal metabolites and resistance mechanisms	[76]
*Bacillus* sp. AP10	*Arabis paniculata*	Genomics	Analyze genes related to plant growth promotion and manganese detoxification	[77]
*Pseudomonas* sp. 2B	*Scutellaria baicalensis*	Metabolomics	Reveal defense-related secondary metabolic reprogramming	[78]
*Guignardia* isolate #1, *Alternaria* #7	*Taxus mairei*	Metabolomics	Accurately pinpoint functionally pertinent strains with successful isolation of endophytes	[79]
*Epichloe festucae*	*Lolium perenne*	Metabolomics	Identify novel metabolites produced by symbiotic endophytic fungus	[80]
*Paraburkholderia*	*Saccharum officinarum*	Metabolomics	Reveal benzoic acid as key signal recruiting nitrogen-fixing endophytes and enhancing associative nitrogen fixation	[81]
*Bacillus subtilis* L1-21	*Citrus* spp.	Metabolomics, Transcriptomics	Reveal mechanisms of surfactin against citrus Huanglongbing through integrated omics analysis	[82]
*Hyphomicrobiales*, *Pteris vittata, Candidatus Saccharimonadaceae*	*Pteris acinosa*	Metagenomics, metabolomics	Reveal arsenic oxidation and speciation mediated by core endophytic microbes in roots	[83]
*Herbaspirillum* sp. G14, *Burkholderia* sp. Y5	*Miscanthus sinensis*	Metagenomics	Reveal diversity and function of diazotrophic root endophytes	[12]
*Enterobacteriaceae*	*Eruca sativa* Mill.	Metagenomics	Reveal core microbiome and resistome	[84]
*Armatimonadete, Verrucomicrobia*	*Populus deltoides*	Single-cell genomics, comparative genomics	Enrich and analyze uncultured root endophytes	[71]

**Table 3:** Compilation of core omics methods resources in plant endophytes research.

Omics	Databases	Applications	Applications	References
Genomics	NCBI GenBank	Genome assembly, identification and functional analysis	https://www.ncbi.nlm.nih.gov/ (accessed on 5 July 2026)	[60,135]
Metagenomics	IMG/M MetaPhlAn 4	Community profiling, structure analysis, and identification of uncultured and uncharacterized species	https://img.jgi.doe.gov/cgi-bin/m/main.cgi (accessed on 5 July 2026) http://cmprod1.cibio.unitn.it/biobakery4/metaphlan_databases/ (accessed on 5 July 2026)	[112,136]
Metatranscriptomics	eggNOG 5.0 UniProt	Functional activity analysis of plant endophytic communities	http://eggnog5.embl.de/ (accessed on 5 July 2026) https://www.uniprot.org/ (accessed on 5 July 2026)	[55,137,138]
Metabolomics	MetaCyc, KEGG	Metabolic profiling and functional metabolite analysis	https://metacyc.org/ (accessed on 5 July 2026) https://www.genome.jp/kegg/ (accessed on 5 July 2026)	[139,140]
Metaproteomics	UniProt, KEGG	Functional protein identification and community activity analysis	https://www.uniprot.org/ (accessed on 5 July 2026) https://www.genome.jp/kegg/ (accessed on 5 July 2026)	[5,138,140]
Single-cell Omics	NCBI GEO, ArrayExpress (EMBL-EBI)	Single-cell heterogeneity analysis and in situ functional characterization	https://www.ncbi.nlm.nih.gov/geo/ (accessed on 5 July 2026) https://www.ebi.ac.uk/biostudies/arrayexpress (accessed on 5 July 2026)	[141,142,143]
Spatial Omics	MGnify + GEO	Spatial localization and in situ interaction analysis	https://www.ebi.ac.uk/metagenomics (accessed on 5 July 2026)	[8,144]

## Data Availability

The data of this study are available from the correspondence author upon reasonable request.
